# 

**Published:** 1889-06-01

**Authors:** 


SATURDAY, JUNE 1st, 1889.

**An American Object Lesson** - 127
**St. John's Hospital for Diseases of the Skin** - 128
**Hydrophobia** - 129
**The Doctor's Pulpit.**—Man and his Enemies - 131
**The Story of the Insane from Year to Year** - 132
**The Archbishop on the National Hospital for the Paralysed and Epileptic** - 133
**Within the Wards.**—A Simple Difficulty - 133
**Notes and News** - 134
**Everybody's Page** - 136
**Annotations.**—A Scandalous School History—Ten Shillings a Child—A Scientist's Appeal to Women - 137
**The Hospital Workshop.**—No. 2. In the Surgery at Guy's 138
**"Her Heart's Mistake."** By E. D. Cumming, Author of "An Ordeal by Silence" - 139
**Turning Over New Leaves.**—The Cause of Cholera—The Nun of Kenmare - 141
**Amusements—Scraps and Gleanings** - 142

EXTRA SUPPLEMENT—THE NURSING MIRROR.

**En Passant.**—A Nurse's Patent—Short Items—A New Mission—Registration of Midwives—A Lady Doctor—Stoke-upon-Trent—Lady Doctors—Deaconesses Institution xxxi
**The Healing Touch** xxxii
**Homes in the Hills** xxxii
**A Wander Round Guy's** xxxiii
**Death in Our Ranks** xxxiii
**Everybody's Opinion.**—The British Nursing Association—Massage—The Food Question—Homes of Rest—Nurses or Water Nymphs—The Age of Nurses xxxiv
**Appointments.—Vacancies** xxxiv
**Examination Questions.—Notes and Queries** xxxiv

### Hospitals urgently needing Annual Subscriptions, Donations and Legacies.

**NATIONAL HOSPITAL FOR THE PARALYSED AND EPILEPTIC**

